# Migration mechanism of grouting slurry and permeability reduction in mining fractured rock mass

**DOI:** 10.1038/s41598-024-51557-y

**Published:** 2024-02-11

**Authors:** Cao Zhengzheng, Wang Pengshuai, Li Zhenhua, Du Feng

**Affiliations:** 1 International Joint Research Laboratory of Henan Province for Underground Space Development and Disaster Prevention, School of Civil Engineering, Henan Polytechnic University, Jiaozuo, 454000 Henan China; 2 Henan Mine Water Disaster Prevention and Control and Water Resources Utilization Engineering Technology Research Center, Henan Polytechnic University, Jiaozuo, 454000 Henan China; 3 Collaborative Innovation Center of Coal Work Safety and Clean High Efficiency Utilization, Jiaozuo, 454000 Henan China

**Keywords:** Coal, Civil engineering

## Abstract

In order to solve the water and gas discharge hazard caused by gob water and harmful gases (such as CO), the method of grouting overburden fractures is adopted to achieve the purpose of safe and efficient mining production in coal mines. This paper carries out the experimental research on the permeability reduction effect of grouting in fractured rock mass, expounds the relationship between gas flow rate and pressure gradient, seepage pressure and permeability, confining pressure and permeability, and analyzes the permeability change law of fractured rock mass before and after grouting. Besides, the grouting migration and permeability reduction model of fractured fine-grained sandstone is constructed by combining grouting test and numerical simulation, which reveals the dynamic evolution law of rock mass permeability in the grouting process. The results show that the permeability of the grouting rock sample decreases from 700–13,000 to 15–300 mD than that of the ungrouting rock sample, and the decrease is more than 95%, which indicates that the sealing performance of grouting slurry is better. Besides, numerical simulations show that the initial permeability of rock samples is 971.9 mD, and the permeability decreases to 45.79 mD after 1800s, and the permeability decreases to 95.3%, which is basically consistent with the experimental results after grouting. The greater the grouting pressure is, the better the grouting effect is. With the increase of the grouting pressure, the increase of the grouting effect is no longer obvious.

## Introduction

With the increasing demand for coal resources in China, the mining of coal seam has been paid more and more attention^1–3^. Once the mining induced fracture zone formed in the process of lower coal mining communicates with the gob area left by upper mining, a large number of gob water and CO and other harmful gases in the gob area can lead to the serious hazard of "water and gas discharge" in coal mine^4–6^. Grouting method can improve the fracture from the micro level, strengthen the stability of rock mass, and realize the safe and efficient mining of coal mine by constructing the "key barrier water and gas layer".

The research on the grouting sealing and seepage reduction of fractured rock mass is gradually developed in order to deal with the water inrush disaster in coal mine. Relevant scholars have carried out a series of researches on the water inrush problem of underground engineering and achieved effective results. Rongchao Xu et al.^7^ studied the influence of strain rate effect on crack stress threshold and revealed the mechanical mechanism of hard rock showing different strength and deformation characteristics at different strain rates. Haiyan Li^8^ developed a visual grouting simulation test system to solve the problem of water gush grouting and plugging in karst pipelines, which realized the real-time observation and image acquisition of slurry diffusion and deposition morphology in the process of dynamic water grouting, and studied the pipe plugging law of cement-sodium silicate quick-setting materials under static and dynamic water conditions. Guangxuan Zhu^9^ carried out the C-S slurry hydrodynamic sealing orthogonal test based on the large-scale fissure hydrodynamic grouting quasi-three-dimensional simulation test platform, and pointed out that the hydrodynamic grouting process was mainly divided into three stages, namely, slurry diffusion, slurry solidified body development and stable solidified body formation. Kaiyu Zhan and Wanghua Sui et al.^10^ carried out orthogonal grouting tests of single fracture plane model under hydrodynamic conditions, studied the influence of flow rate and slurry gel time on water plugging effect, and clarified plugging mechanism of grouting slurry under hydrodynamic conditions. Through the quasi-three-dimensional hydrodynamic grouting model test, Shucai Li^11^ put forward the U-shaped diffusion law of slurry and the stratified and partitioned diffusion mechanism of cement slurry, and pointed out that the effect of cement slurry grouting and water plugging depends on the diffusion range of deposition and retention core, and obtained the rapid water precipitation deposition principle of slurry and the diffusion law of deposition and retention core. Cong Zhang et al.^12^ realized the simulation of power-law fluid pulsating grouting through the pulsating penetration grouting simulation test device, obtained the percolation and diffusion patterns of power-law fluid under different pulsating construction parameters, and concluded that the spatial distribution of viscosity is an important factor affecting the sealing of grouting slurry. Renshu Yang et al.^13^ used CT scanning technology to observe and study the fracture change of the specimen after grouting, and analyzed the change law of the fractal dimension of the internal fracture of the specimen, so as to analyze the grouting effect. By testing the permeability before and after grouting, Yue Yu^14^ analyzed and discussed the grouting effect, and intuitively gave the grouting effect of different viscosity grouts for rock samples. Based on the self-developed large triaxial seepage flow-grouting multifunctional test platform, Qi Liu et al.^15^ carried out variable mass seepage flow test and studied the improvement of the impermeability stability of the filling medium in the solution cavity by grouting. Jianping Wei et al.^16^ constructed a variable mass seeping model for plugging and lowering the permeability of fractured coal by grout, which was verified by comparing the permeability test of fractured coal samples before and after grouting. The established model can better reflect the influence of slurry particle deposition process on coal fracture structure and permeability. Jing Yang^17^ carried out the field test work of grouting and plugging of water-conducting fractures in overlying rock through horizontal directional drilling on the ground, and obtained that the optimal grouting horizon for plugging mining-induced fractures is the closest rock layer with hard lithology below development critical value of water-conducting fractures in overlying rock and the determination criterion.

The above research explains the sealing mechanism of grout for fractured rock mass and analyzes the grouting effect through tests^18–21^. However, the mechanism of grouting slurry migration and permeability reduction in mining fractured rock mass has not been revealed at present. Since the dynamic process of grout sealing in fractured rock mass cannot be directly observed, and the macroscopic test effect is mainly manifested in the permeability, therefore, this paper combines the grouting effect test and numerical simulation to study the permeability characteristics in the grouting process. Through comparative analysis, a better grouting treatment method is optimized.

## Effect test of grouting infiltration reduction

### Basic principles

#### Split test system

The rock samples are obtained from Tongxin coal mine in Datong mining area and processed into fine-grained sandstone samples with standard sample size of Φ50 mm × 100 mm in the laboratory. Under the influence of mining, rock strata fracture occurs, resulting in the collapse of rock strata, and the fractures formed are the main channels of "water and gas discharge". Therefore, Brazil splitting is used to simulate the fracture phenomenon of field rock strata in complete rock samples^22^.

**Figure 1:**
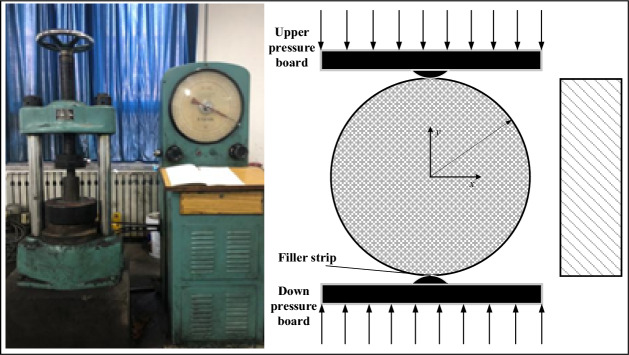
Splitting system and schematic diagram.

**Figure 2:**
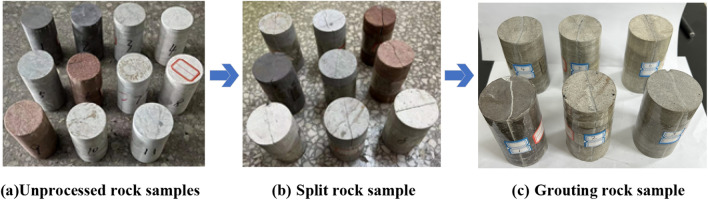
Preparation of rock samples.

The 60 tons ordinary press in Henan Polytechnic University is used in the test, and the test system is mainly composed of instrument panel, manual controller, hydraulic transmission, pressure source and other components, shown in Fig. 1. This test is mainly to achieve the effect of rock sample fracture through the similar simulation of the fracture structure plane formed after the fracture of the rock layer. The rock samples before and after processing are shown in Fig. 2.

#### Grouting test system

The test system adopts the self-developed rock grouting test system, which is mainly composed of seven parts, containing air pump, hydraulic manual pump, pressure regulator, grouting tank, control and data acquisition system, model grouting chamber and measurement system. The grouting test of fractured rock mass can be carried out under different grouting pressure and different confining pressure. The overall size of the grouting test bed is 2 m × 1 m × 2 m (length × width × height), and the model grouting chamber is a cylinder of Φ55 mm × 120 mm. The grouting chamber is composed of stainless steel except for the slurry inlet and outlet connected by a soft rubber tube. The left and right ends of the grotching chamber are all connected with the main body, and the left end is equipped with an axial displacement loading device, which can change the axial displacement according to the model size and model change. The main body is connected to the side of the hydraulic hand pump, which can control the confining pressure of the rock sample through this device, and can apply the maximum confining pressure of 10 MPa. The data collected by this system can be used to measure time, gas flow rate, grouting pressure, confining pressure and slurry flow rate.

#### Penetration test system

The penetration test system mainly includes air intake system, thermostat system, vacuum degassing system, air outlet acquisition system and data acquisition and processing system. Using this test system, the influence law of confining pressure, axial pressure and gas pressure on sample permeability can be accurately and reasonably analyzed, so as to evaluate and analyze rock permeability.

### Test conditions

The splitting test uses direct loading of rigid pad, called flat plate splitting method, and radial load is applied to the rock sample at a loading rate of 0.5 kN/s until the rock sample splits. The mass percentage of each component in slurry is set. Specifically, fly ash: general silica cement: loess accelerator: expansion agent: bentonite: water reducing agent: curing agent = 55%: 28%: 12.2%: 3%: 0.7%: 0.8%: 0.6%: 0.7%, water–solid ratio W/S = 1:1. The grouting test grout is selected with the above proportioned grout, the grouting pressure and confining pressure are selected as 3 MPa, the grouting time is selected as 30 min, and the test data are recorded after the grouting is completed. Five different confining pressures (Cs = 2,4,6,8,10 MPa) are set in the penetration test, and six different inlet pressures (Ps = 0.3, 0.6, 0.9, 1.2, 1.5, 1.8 MPa) are set for each confining pressure. In the penetration test, the rock sample is saturated with water, the vacuum degaging system is opened, and then a certain axial pressure is applied to the rock sample. After the axial pressure is stable, a certain value of confining pressure is applied, the nitrogen bottle is opened, and the fluid driving pressure is adjusted to a certain value. After the stability, the valve is opened for testing, and the data results are finally recorded. The data acquisition system is mainly composed of a desktop computer, a precision balance and a water container. Real-time data collection and analysis can be carried out during the experiment. The experiment mainly collectes the data of gas flow, seepage confining pressure, air pressure and permeability before and after grouting. By analyzing the relationship between gas flow and pressure gradient, seep confining pressure and permeability, and seep air pressure and permeability, the influence of grouting on the permeability characteristics of fractured rock mass is obtained.

### Results analysis

In order to explore the grouting effect of grouting on fractured rock samples, two fine-grained sandstone rock samples are selected before and after grouting, and permeability characteristics are compared and analyzed.

#### Analysis of the relationship between gas flow and pressure gradient

**Figure 3:**
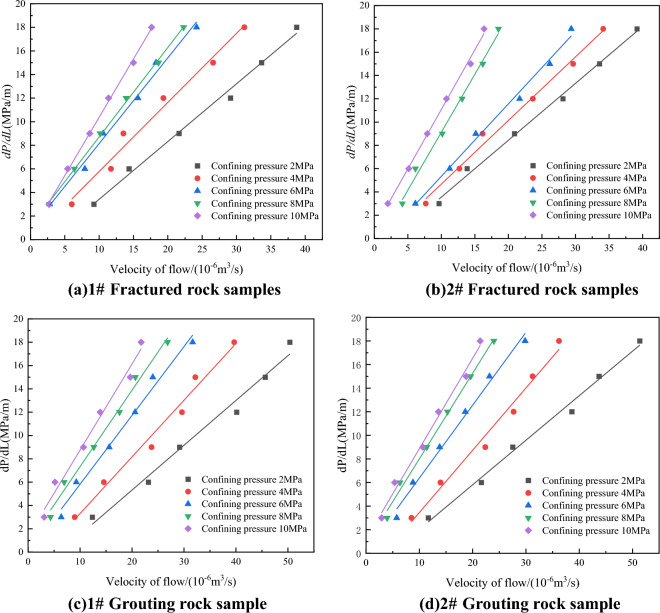
Fitting curves of flow and pressure gradient under different confining pressures.

Figure 3 shows the correlation between the gas flow and the pressure gradient after the splitting test of different rock samples in the process of percolation test. The curves are fitted by linear equations, and the fitting coefficients R^2^ are all above 0.99, indicating a good degree of fitting. As can be seen from Fig. 3, the gas flow of rock samples before and after grouting shows an obvious downward trend with the increase of confining pressure, which is because the increase of confining pressure narrows the fracture channel, resulting in the decline of gas flow. The gas flow of fractured rock mass is 21.0–35.7 ml/s, and the gas flow of grouting rock mass is 2.9–51.4 ml/s.

#### Analysis of the relationship between seepage pressure and permeability

For the gas seepage test in rock samples, it is generally believed that it conforms to Darcy’s law. According to the gas flow rate of rock samples tested in the test and the pressure at both ends of the rock samples, the permeability of rock samples can be calculated. The formula is as follows,

1 $$ K = \frac{{2QP_{0} \mu L}}{{\left( {P_{1}^{2} - P_{2}^{2} } \right)A}}, $$

where *K* is the permeability, the unit is mD;* Q* is the gas seepage flow rate, the unit is cm^3^/s; *P*_*0*_ is the standard atmospheric pressure, the unit is MPa; *μ* is the dynamic viscosity of nitrogen, the unit is Pa·s; *L* is the length of the specimen the unit is cm; *P*_1_ is the inlet pressure of the specimen the unit is MPa; *P*_2_ is the outlet pressure of the specimen, which is 0 MPa; *A* is the effective penetration area of the specimen, the unit is cm^2^.

**Figure 4:**
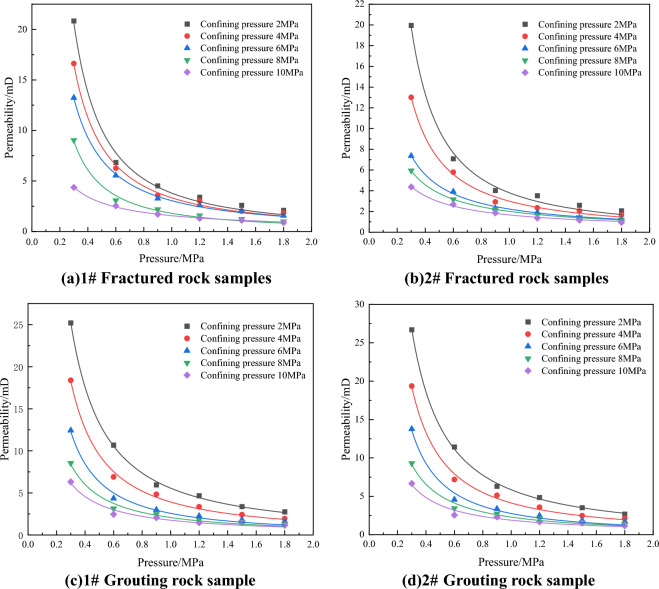
Fitting plot of the relationship between seepage pressure and permeability under different confining pressures.

Figure 4 shows the fitting diagram of the relationship between permeability and seepage pressure of rock samples before and after grouting. The curve is fitted by a power equation, and the fitting coefficient R^2^ is above 0.99, indicating a good degree of fitting. It can be seen from Fig. 4 that with the increase of gas pressure, the permeability of rock samples before and after grouting shows a significant downward trend. This is because according to the permeability calculation formula, the percolation gas pressure affects the denominator, and the increase of pressure difference leads to the increase of denominator and the corresponding decrease of permeability value, indicating that the permeability of rock samples is mainly affected by gas pressure, that is, gas pressure is the dominant factor of rock sample permeability.

#### Analysis of relationship between confining pressure and permeability

**Figure 5:**
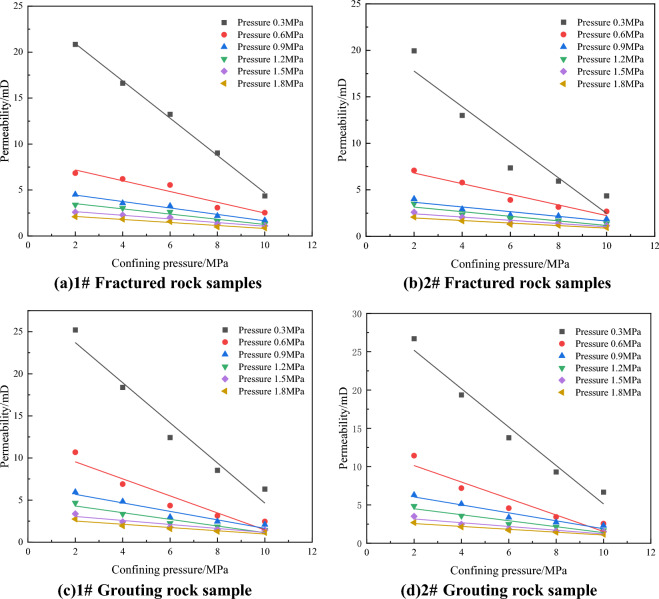
Fitting diagram of the relationship between confining pressure and permeability under different air pressures.

Figure 5 shows the fitting diagram of the relationship between seepage confining pressure and permeability of rock samples before and after grouting. The curves are fitted by linear equations, and the fitting coefficients R^2^ are all greater than 0.9, indicating a good degree of fitting. It can be seen from the Fig. 5 that with the increase of the seeping confining pressure, the permeability tends to decrease, which is because the increase of the confining pressure reduces the area of the gas channel, the opening of the fracture further decreases, and the permeability decreases. The permeability values of fractured rock samples range from 0.9 to 20.8 mD, and the permeability values of grouting rock samples range from 1.1 to 26.7 mD.

#### Comparative analysis of permeability before and after grouting

**Figure 6:**
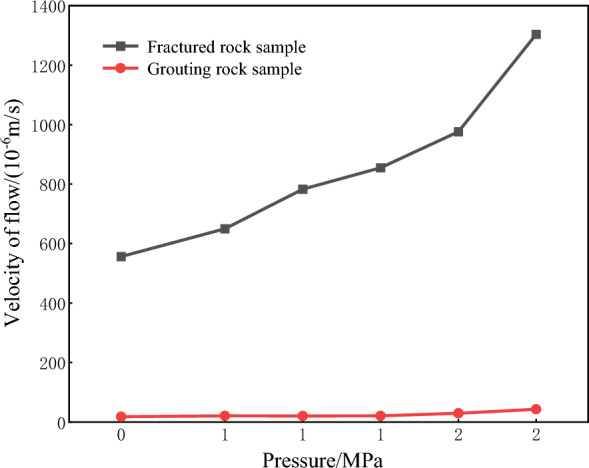
Comparison curves of seep flow test results before and after grouting of fine-grained sandstone rock samples.

The reliability of the grouting slurry is verified by comparative analysis of the permeability characteristics of the fine-grained sandstone samples before and after grouting. Figure 6 shows the grouting of the fractured rock sample of fine-grained sandstone. After grouting, the gas flow rate of the rock sample decreases greatly compared with that of the fractured rock sample, the overall flow rate is 15–40 × 10^–^6 m^3^/s, while the gas flow rate of the ungrouting fractured rock sample is 500–1400 × 10^–^6 m^3^/s, the decrease rate is more than 95%, which indicates that the slurry has better sealing performance and can reduce the gas flow rate better. Thus, the gas flow rate is reduced.

**Figure 7:**
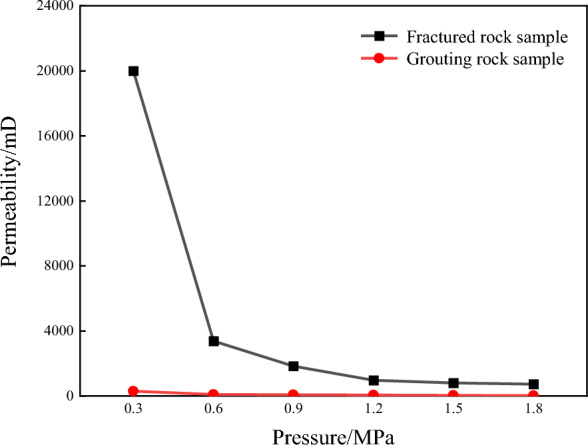
Comparison of permeability test results of fine-grained sandstone samples before and after grouting.

The permeability comparison curve of fine-grained sandstone before and after grouting is shown in Fig. 7. After grouting, the gas flow velocity of the rock sample is still larger than that of fractured rock sample, the overall value is in the range of 15–300 mD, while the permeability of the ungrouting fractured rock sample is 700–13,000 mD, the decrease is more than 95%, indicating that the grout has better sealing performance, reduces the permeability of the grouting rock mass, and can slow down the gas penetration ability. Thus, the sealing of grouting strata is improved.

## Numerical simulation analysis of grouting infiltration reduction in fractured rock mass

The model tests are numerically simulated using the physics module of Comsol Multiphysics software. Due to the complexity and invisibility of geological conditions, the process of percolation diffusion and down-infiltration of grouting slurry in the fracture is a process of continuous deposition of grout particles under the action of external stress and self-gravity, and it is difficult to describe the dynamic evolution of permeability of grout in the process of migration in the fracture rock sample. Therefore, the mathematical model of grouting slurry transport and permeability reduction in fractured rock mass is established. Combined with the physical parameters of grouting slurry, a numerical model is established to study the dynamic evolution law of permeability in the process of grouting slurry transport in fractured rock mass.

### Data model establishment

#### Hypothesis model

**Figure 8:**
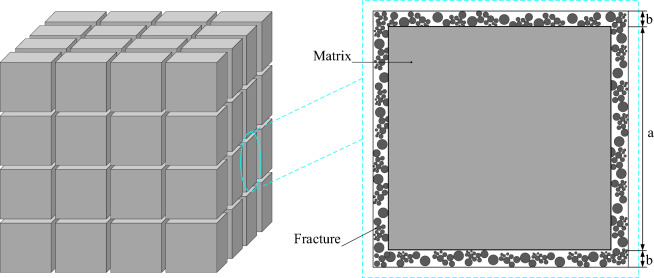
Structure diagram of a micro-element.

For the grouting of fractured rock mass, the slurry flows along the direction of the fracture and penetrates in the direction of the rock matrix. Under the influence of fluid dynamics, the particles in the slurry deposit, causing the pore fracture of the rock mass to narrow until it is blocked. The macroscopic appearance of the rock mass shows a decrease in porosity and fracture rate, and the permeability is reduced, resulting in the effect of “plugging”. There are micro-fractures in the rock mass itself. Under the influence of mining, fluid seawage-migration and other factors, the development and expansion of micro-fractures gradually form cracks of different lengths and sizes until they form through water and gas conduction channels, causing disasters such as water inrush and gas discharge hazard. The fracture is an important factor affecting the permeability properties of the rock mass, and also becomes the main channel of the grouting slurry seeping diffusion. The grouting effect is reflected in the sealing effect of the grouting slurry on the fracture, namely, the change of permeability. The influence of the rock matrix on the slurry seepage is ignored, shown in Fig. 8. It consists of a cubic matrix of side length a and a fissure of width b/2 around the matrix.

In this model study, the following assumptions are made.

1. The deposition of slurry particles does not affect the flow pattern of slurry.
2. The slurry particles are incompressible and keep the injection concentration unchanged.
3. The deposition process of suspended particles in the slurry is irreversible, and once deposited, it is not separated.
4. The slurry is continuous in the process of movement and satisfies continuity equation.

#### Geometric modeling and meshing

**Figure 9:**
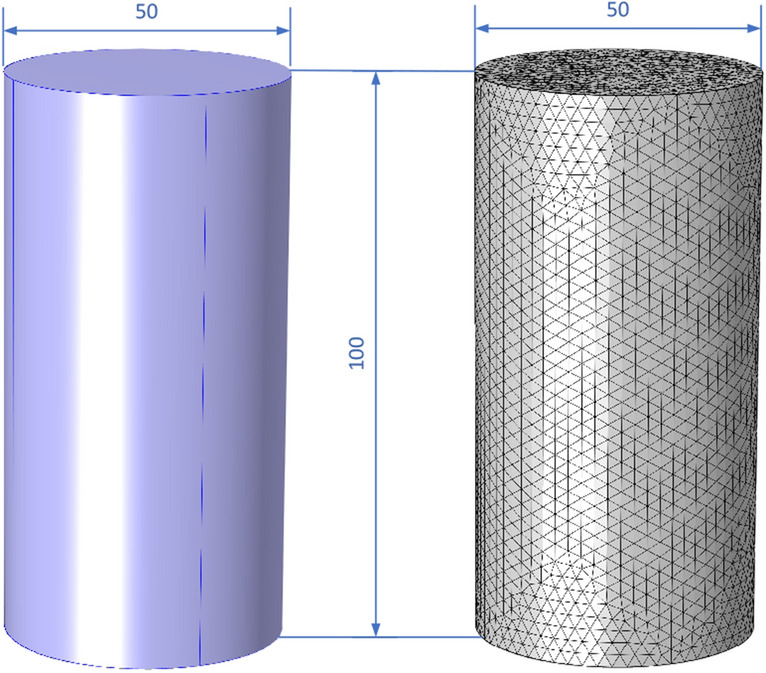
Geometric model and mesh model.

To simplify the calculation, the geometric model of the numerical model is constructed by the fine-grained sandstone fractured rock sample for detailed study, shown in Fig. 9. The geometric model is a 50 mm × 100 mm cylinder with one domain and three faces. The model size is consistent with the real object. The mesh model is a free tetrahedral mesh, and the mesh size is selected to be refined to ensure accuracy. The complete mesh contains a total of 179,263 domain elements.

#### Geometric model assignment

**Figure 10:**
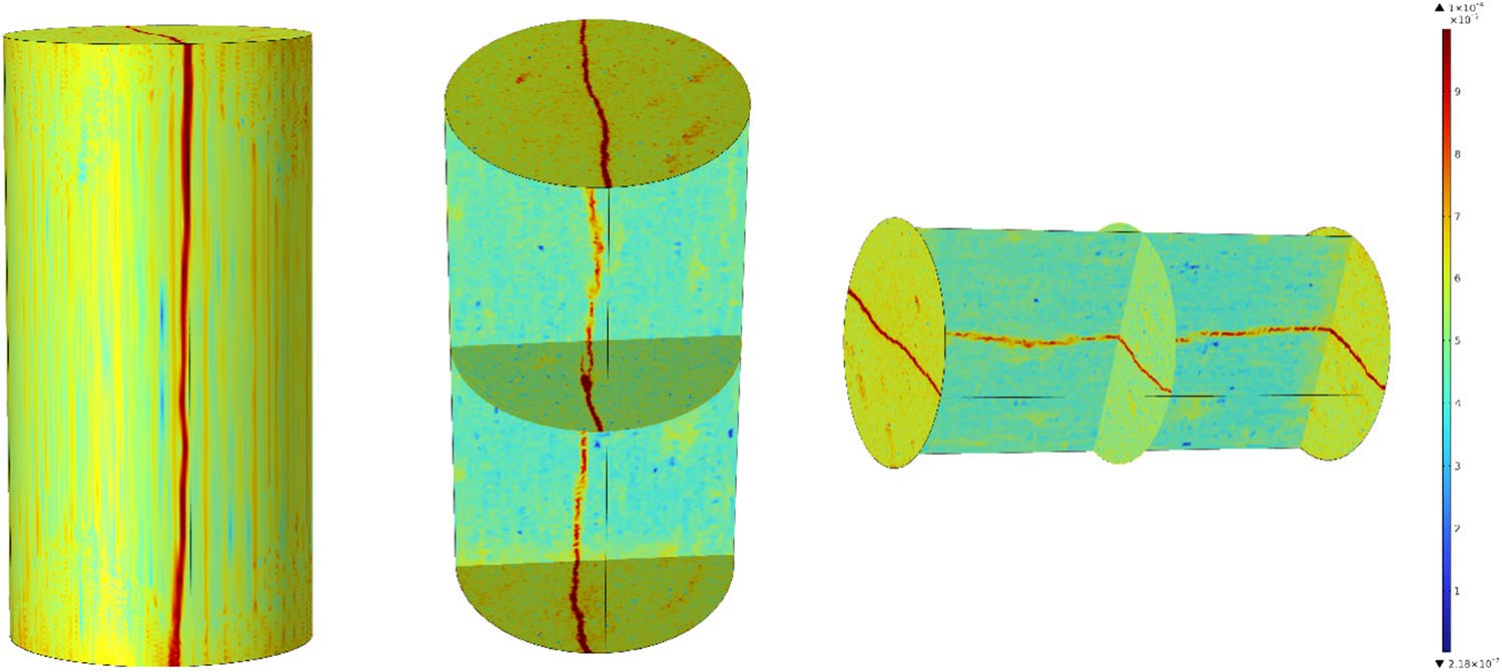
Cloud map of gap distribution.

The geometric model construction is the gray value extracted by MTLAB, between 0–1, and the generated interpolation function contains the structure of the fractured rock sample, which can be imported into COMSOL Multiphysics. It is named int1 (*x*, *y*, *z*), and the darker the colour is, the closer the value is to 1. The distribution cloud diagram of the fissure is shown in Fig. 10.

For the constructed geometric model, the initial fracture width, fracture fraction and permeability need to be assigned for numerical simulation calculation, then

2 $$ {\text{b}}_{0} = 2^{ - 4} \cdot {\text{int}} (x,y,z), $$

3 $$ \varphi_{0} = 3\frac{{b_{0} }}{a}, $$

4 $$ k_{0} = \frac{{b_{0}^{3} }}{3a}. $$

#### Initial and boundary conditions

For the physical module of the finite element software COMSOL Mutiphysics, the boundary conditions can be set with fixed values or variables that change with time. The setting of the boundary conditions should be combined with the existing test conditions to ensure that the accuracy of the theory and numerical simulation can be verified by the test. Therefore, according to the existing test conditions, the numerical model should satisfy the following initial and boundary conditions.

- (1) The formula of slurry viscosity curve measured by the ratio of grouting material is,
  5 $$ y = 1.031x^{2.36} + 1579.08. $$
- (2) The upper boundary of the model is the inlet boundary, and the grouting pressure is 3 MPa. The lower boundary of the model is the outlet boundary and the pressure is 0 MPa.
- (3) No flux boundary is set around the model, namely, the slurry does not seepage through the surrounding boundary. The influence of confining pressure on the numerical model is mainly realized by the assignment of the initial crack width.
- (4) The upper surface of the model is set as the initial concentration boundary of the grouting slurry.
- (5) The simulation time is set as 30 min, which can be extended appropriately.

### Governing equation

#### Slurry flow equation

The slurry moves in the fractured rock mass, and the fluid velocity and pressure gradient are assumed to satisfy linear Darcy laminar flow. The Darcy equation is expressed by Eqs. (6) and (7).

6 $$ \nabla \cdot \left( {\rho u} \right) = Q_{m} , $$

7 $$ q = - \frac{k}{\mu }\nabla p, $$

where *k* is the permeability; *p* is fluid pressure; *ρ* is the fluid density; *Q*_*m*_ is the source and sink term; *q* is Darcy velocity; *μ* is the dynamic viscosity.

#### Mass conservation equation

Since the solid particles in the grout are incompressible, the particle density is constant, and the conservation equation of grout mass in fractured rock mass satisfies the equation

8 $$ \frac{\partial }{\partial t}\left( {\varphi C} \right) = \nabla \left( {\varphi C\overrightarrow {v} } \right) - CK_{dep} . $$

*K*_*dep*_ is the slurry particle deposition coefficient, which satisfies the equation

9 $$ K_{dep} = \frac{{3\left( {1 - \varphi_{0} } \right)v}}{{2b_{0} }}\mu , $$

where *φ*_*0*_ is the initial crack rate; *φ* is the fracture fraction; *b*_*0*_ is the initial crack width; *C* represents the particle volume fraction within the fissure.

#### Permeability evolution equation

The grouting slurry particles in the fractured rock mass gradually enter the deep part of the fractured rock mass under the action of percolation and deposition, and the fractured channel is gradually blocked, and the macroscopic porosity and permeability of the fractured rock mass are reduced. According to the Kozeny-Carman equation, the relationship between permeability *K* and fracture fraction *K*_*0*_ can be established.

10 $$ \frac{K}{{K_{0} }} = \left( {\frac{\varphi }{{\varphi_{0} }}} \right)^{3} \left( {\frac{{1 - \varphi_{0} }}{1 - \varphi }} \right)^{2} = \frac{{\left( {\rho_{s} \varphi_{0} - S} \right)^{3} \left( {1 - \varphi_{0} } \right)^{2} }}{{\varphi_{0}^{3} \left( {\rho_{s} + C} \right)\left[ {\rho_{s} \left( {1 - \varphi_{0} } \right) + C + S} \right]^{2} }}. $$

According to the above equation, the ratio of the permeability of the fractured rock sample to the initial permeability can be obtained by solving *S* and *C*. According to the classical particle migration-deposition model^23–26^, the relationship between the concentration of deposited particles *C* and the concentration of suspended particles *S* can be established.

11 $$ \frac{\partial S}{{\partial t}} = \frac{{K_{dep} \varphi C}}{{\rho_{b} }}, $$

12 $$ \rho_{b} = \left( {1 - \varphi_{0} } \right)\rho^{\prime}_{s} + \left( {\varphi_{f0} - \varphi } \right)\rho_{s} , $$

where *ρ*_*b*_ is the dry volume density of fractured rock mass; *ρ*_*s*_*’* is the particle density of fractured rock mass. The above equation can be simplified into

13 $$ \frac{{\rho_{s}^{2} }}{{\rho_{s} \varphi_{0} - S}}\frac{\partial S}{{\partial t}} - \rho_{s} \frac{\partial S}{{\partial t}} = K_{dep} C. $$

Combined with the above equations, the mechanical model of grouting slurry migration and permeability reduction in fractured rock mass is constructed.

### Calculating parameters

The calculated parameters of the numerical simulations are shown in Table 1. In order to study the influence of grouting pressure on the grouting effect, the grouting pressure is divided into four grades, namely, 1 MPa, 2 MPa, 3 MPa and 4 MPa.

**Table 1:** The parameters calculated by numerical simulation.

Density of slurry/(kg·m^3^)	Grouting pressure/MPa		Density of water/(kg·m^3^)	Viscosity of water/(Pa·s)
	Inlet	Outlet		
1340	1/2/3/4	0	1000	0.001

### Results analysis

#### Dynamic evolution analysis of permeability

**Figure 11:**
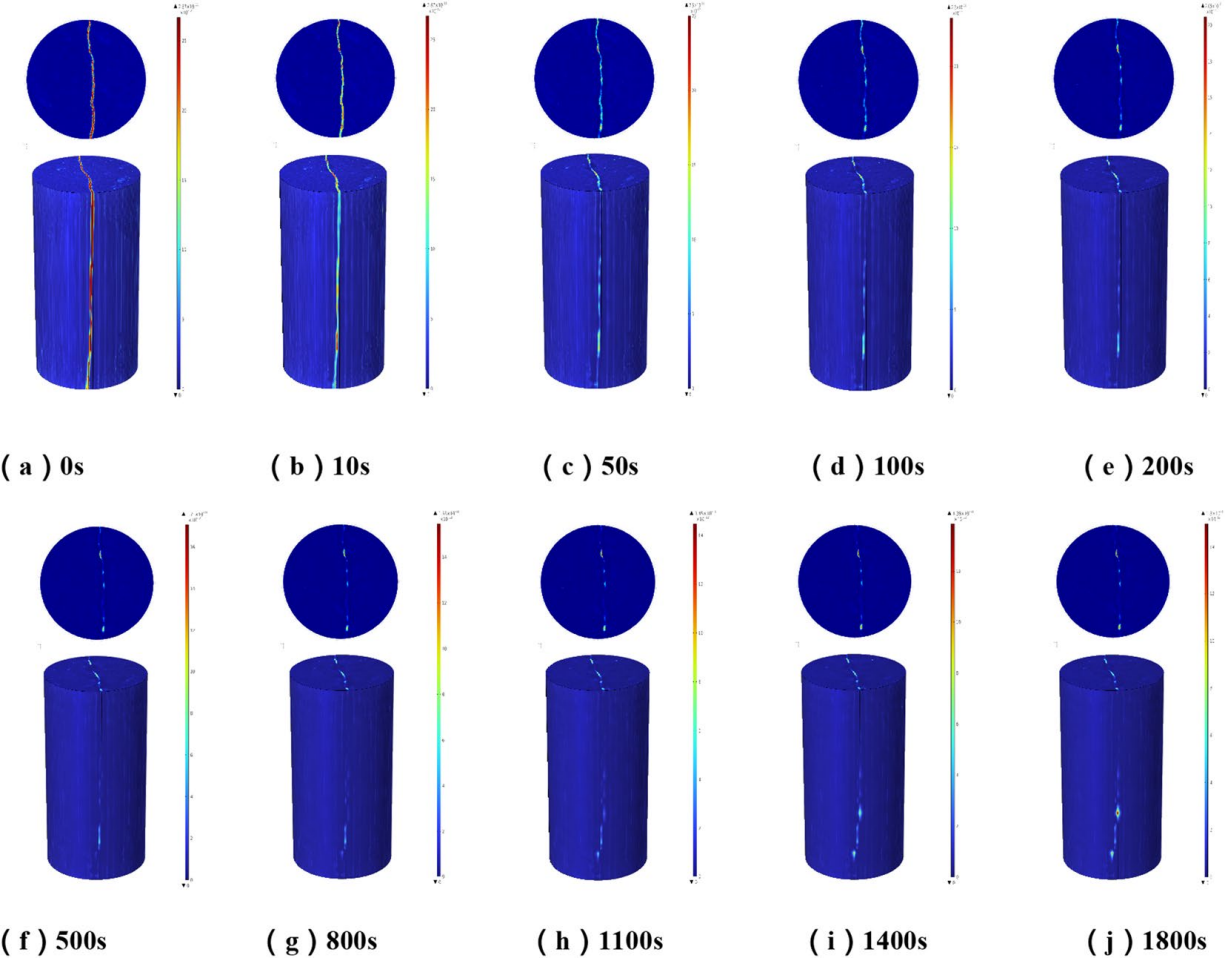
Cloud map of the permeability change of the whole and section of the rock sample at different times.

Figure 11 shows the permeability cloud maps of the whole numerical simulation of the rock sample and its middle section at different times. From the overall rock sample, the permeability is mainly distributed along the direction of the fracture, and the colour close to red represents the greater permeability. After the grouting begins, the colour of the fracture on the permeability cloud map gradually changes from red to orange until it is close to the dark blue on both sides of the fracture, which indicates that chemical reactions and deposition of slurry particles begin to occur during the slurry seeping along the fracture, resulting in the decrease of permeability and the continuous change of fracture colour with time. With the increase of grouting time, the colour change speed changes from fast to slow, and the colour change shows uneven phenomenon, this is because the initial grouting, the crack width is large, the slurry under the grouting pressure drive, rapid seeping and diffusion.

When the fracture is filled, the deposition of particles decrease the width of the fracture and the permeability, and the slurry percolation speed slows down. Due to the roughness of the fracture surface, the opening degree of the fracture is not a fixed value, so there are some locations where the slurry particles deposit faster and the permeability decreases faster, while some locations where the slurry particles deposit slower and the permeability decreases slower, and finally the colour is uneven. The maximum permeability is almost unchanged in the first 50 s, because the slurry first fills the fissure space, no deposition or less deposition occurs, but as time increases, the slurry particle deposition increases, and the maximum permeability begins to decrease. Therefore, the grouting time can be appropriately extended in the project to increase the grouting effect. In order to research the process of permeability change inside the rock sample, the transverse section of the geometric model at the middle position is selected. At the beginning of grouting, the colour of the fracture gradually became lighter, and the colour on the fracture shows a staggered phenomenon of orange and red. With the grouting in the 1800s, most of the locations on the fracture are close to the colour of the rocks on both sides of the fracture, indicating that the permeability decreases significantly. However, there are still a small number of positions with light colour, namely, relatively large permeability. This is because of the different fracture width and slurry particle deposition speed. Therefore, on the same transverse section, although the permeability reduction is different, it shows a decreasing trend. In the first 10 s of grouting, the maximum permeability on the slice decreases slowly, in the 10–1100 s, the permeability decreases rapidly, and the change is small in the 1100–1800s, which indicates that the grout sealing of the fracture has experienced three stages. At the stage of grouting filling, the slurry seepage diffuses to all parts of the fissure, with less deposition and mainly seep diffusion. At the stage of flow and deposition, the slurry particles begin to deposit and gradually block the fissure, and the permeability decreases significantly. At the stage of plugging, with the increase of grouting time at this stage, a small amount of slurry particles would still be deposited and the permeability would also decrease, but the decrease is small. It can be seen that extending the grouting time has limited effect on the grouting effect.

The domain probe is set in COMSOL Mutiphysics, and the expression is set to permeability. After calculation, the change of permeability of fractured rock samples in 0–1800s could be derived after data processing. The evolution curve of permeability of rock samples under different grouting pressures is obtained. Because the initial fracture space is large, the slurry flow rate is fast, and the slurry particles are deposited quickly. With the deposition of slurry particles, the fracture space is gradually reduced, the slurry flow rate is reduced, and the slurry is difficult to percolate and diffuse. The permeability is basically unchanged and the change process is similar to the change of permeability cloud map. The initial permeability of the fractured rock samples is 971.9 mD, which decreased to 45.79 mD in 1800s, and the permeability decreased to 95.3%. This result is basically the same as the decrease of permeability in the grouting test, which indicates that the numerical model is accurate and verifies the reliability of this numerical simulation.

#### Analysis of grouting pressure on grouting effect

**Figure 12:**
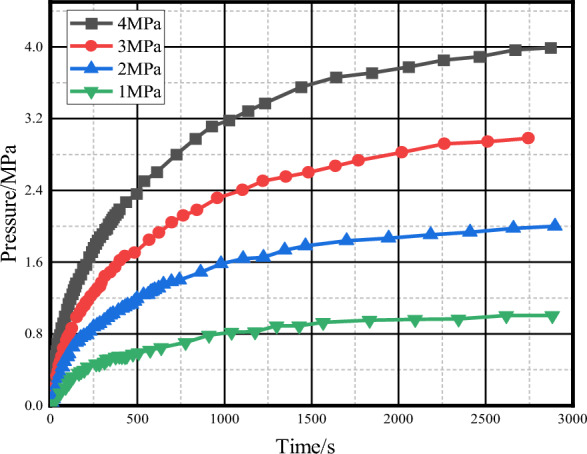
Pressure curve in the fracture hole of rock sample with time under different grouting pressure.

Figure 12 shows the curve of the pressure in the fracture hole as a function of the grouting time during the grouting process of the geometric model of the fractured rock sample of fine-grained sandstone. The pressure in the fracture hole changes dynamically with time. For different grouting pressures, with the increase of grouting time, the pressure in the fracture hole of the fractured rock sample increases continuously. When the time is long enough, the grouting pressure can be reached, but the larger the grouting pressure is, the faster the pressure in the fracture hole increases, and the time required to reach the grouting pressure is extended. It shows that the higher grouting pressure can be used to increase the pressure in the fractured rock sample hole rapidly in a short time.

**Figure 13:**
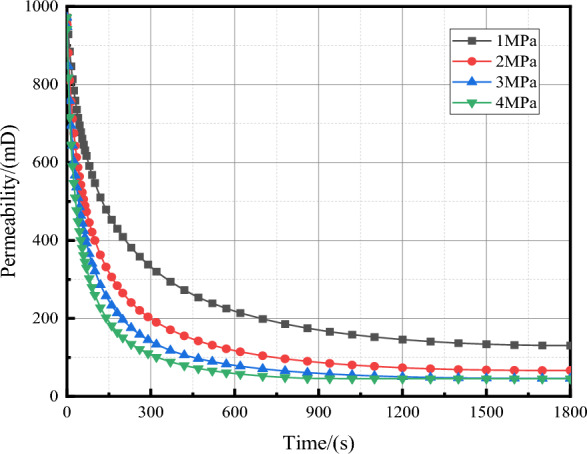
Plot of permeability of rock samples with time under different grouting pressures.

It can be seen from Fig. 13 that the same fine-grained sandstone fractured rock sample has the same structure. With the increase of grouting time, the permeability shows a law of rapid decline, and then slow decline until it is almost constant. The larger the grouting pressure is, the faster the permeability decreases in the early stage of grouting. This is because the grouting pressure is the main force of slurry seepage, while the slurry diffusion is mainly the effect of concentration difference, and the change of slurry transport speed is mainly the effect of grouting pressure, which indirectly affects the sedimentation speed of slurry particles, and finally shows that there is a difference in the decrease speed of permeability speed. The influence of grouting pressure on the final grouting effect is obviously different. When the grouting pressure is 1 MPa, the final permeability is 130 mD, and the permeability decreases by 86.6%. When the grouting pressure is 2 MPa, the final permeability is 66.4 mD, and the permeability decreases by 93.2%. The decrease of permeability is more than 95%, which indicates that the effect of grouting is gradually enhanced with the increase of grouting pressure, but the grouting pressure is not linear relationship with the grouting effect. When the grouting pressure reaches a certain value, the influence of increasing the grouting pressure on the final grouting effect is small, such as the grouting pressure of 3 MPa and 4 MPa. The final permeability decline value is close, indicating that the choice of grouting pressure is not better, but higher grouting pressure can quickly reduce the permeability, and the grouting distance also increases. Therefore, when the grouting time is short, the grouting pressure can be increased to obtain better grouting effect.

## Conclusions

1. The gas flow rate, pressure gradient, volume flow velocity, and pressure gradient show obviously different characteristics in the seepage experiment of fractured rock samples, which can be described by linear equation fitting. The seepage pressure and permeability show obvious nonlinear characteristics, which can be described by power equation. With the increase of seepage confining pressure and seepage pressure, the permeability of rock samples decreases gradually. The initial value of the permeability of fine-grained sandstone is 970.2 mD, and it is 46.8 mD after grouting, and the permeability decreases by 95.3%.
2. Numerical simulation is used to study the influence law of different grouting pressure on the grouting effect and reveal the evolution law of permeability in the process of grout migration. The grout migration in the fractured rock mass has experienced three stages, containing filling and diffusion stage, seepage and deposition stage, and plugging stage. The initial permeability of numerical simulation is 971.9 mD, and the permeability after 1800s is 45.79 mD, and the grouting effect reaches 95.3%.
3. The geometric model is consistent with the real fracture structure, and the numerical model structure is accurate, which verifies the feasibility of the mathematical model of grouting slurry migration and permeability reduction of fractured rock samples. Under different grouting pressures, the pressure in the fracture hole of fine-grained sandstone reaches the grouting pressure. The greater the grouting pressure is, the better the grouting effect is. However, with the continuous increase of the grouting pressure, the grouting effect gradually weakens, and the optimal grouting pressure is 3 MPa.

## Data Availability

Some or all data, models, or codes generated or used during the study are available from the corresponding authors by request.
